# Book Reviews

**Published:** 1800-04

**Authors:** 


### CRITICAL RETROSPECT

### OF

### MEDICAL AND PHYSICAL LITERATURE.

### [FOREIGN AND DOMESTIC.]

---

*Principles of Modern Chemiſtry, ſyſtematically arranged by Dr.* FREDERICK CHARLES GREN, *late Profeſſor at Halle in Saxony. Tranſlated from the German; with Notes and Annotations concerning later Diſcoveries, by the Tranſlator, and ſome neceſſary Tables. Illuſtrated by plates.* 8vo. Vol. I. pp. 448, and ſix plates. Vol. II. pp. 487, a double plate of the modern chemical characters, a variety of uſeful Tables, and a copious Index. Price 18s. 1800. *London.* Cadell and Davies.

AT length, we have the ſatisfaction to announce the appearance of this ineſtimable work, in an Engliſh dreſs.* The celebrated author, whoſe memory will ever be dear to the friends of chemical ſcience, has beſtowed a conſiderable portion of his active and uſeful life on the improvement and completion of this elementary Treatiſe, which, independent of his other works in various branches of learning, aſſigns to him an honourable place among modern writers.

We fully agree with the tranſlator, that the preſent volumes compriſe an elaborate and ſatisfactory abſtract of the author's "*Syſtem of Chemiſtry,*" which appeared in 1794 at Halle, in four large volumes—the moſt complete and ſyſtematical work ever publiſhed on this ſcience.

Without entering into an inveſtigation of the theory which the learned author has adopted in Natural Philoſophy, we ſhall in this place only obſerve, "that, in the Syſtem of Chemiſtry above mentioned, he did not ſo much adhere to the old *phlogiſtic ſyſtem,* as was objected by ſome, but rather that he has framed a ſyſtem of his own, which he called *ecclectic,* though he there explains every phenomenon, not only according to his own doctrine, but alſo *hiſtorically,* according to that of both the *phlogiſtian* and *antiphlogiſtian* philoſophers.—To that ecclectic ſyſtem the author adhered till his death; but the reader is requeſted to obſerve, that in the preſent work Dr. Gren has merely ſtated the grounds of his favourite chemical creed, and continued throughout the reſt to explain all the phenomena treated of according to the *modern antiphlogiſtic ſyſtem,* in the ſtricteſt ſenſe."

We

---

* Vide our Journal, Vol. I. pp. 206 and 514

We have extracted this declaration from the tranſlator's inſtructive preface, in which he alſo gives an account of his labours, to render this work as extenſively uſeful as the preſent ſtate of ſcience will admit. With this laudable intention he has, 1. Studiouſly retained the references in the paragraphs, or rather increaſed their number, in order to aſſiſt the recollection of the reader. 2. He has added, in notes, all ſuch diſcoveries and improvements made ſince the German publication of this work, as come within the compaſs of an elementary treatiſe. 3. The *new nomenclature* of the French chemiſts has, with a few judicious variations, been preferred, tho' at the beginning of the work the *ancient terminology* has purpoſely been employed; yet, after having explained every particular ſubſtance and its conſtituent parts, the new names are ſucceſſively introduced, and then, for ſome time, both have been promiſcuouſly uſed, ſo that, towards the latter part, the preference has almoſt uniformly been given to the new nomenclature. 4. The moſt neceſſary and uſeful chemical inſtruments are repreſented in ſix elegant engravings, by the maſterly hand of Lowry, and a ſeventh plate is added to the ſecond volume, exhibiting a ſufficient number of ſpecimens of the new chemical ſymbols, progreſſively from the primitive and ſimple to the compound. 5. The induſtrious editor and tranſlator of this work has farther enhanced its value by an appendix rarely to be met with in ſimilar attempts; as it contains *Tables of Affinities*, the new *Chemical Symbols*, the *ſpecific and abſolute Gravity of Bodies*, the compariſon of *Fahrenheit's* with *Reaumur's* Thermometer, the former *French Weights and Meaſures*, as well as the preſent *Metre, Litres*, and *Grammes*, together with thoſe of the Engliſh—to which he has added a ſmall *Chemical Library*. The whole is concluded with a copious *Alphabetical Index*, to facilitate occaſional reference, and chiefly intended to ſerve as a compendious Dictionary of both the old and new Nomenclature.

We refrain from ſaying more, with reſpect to this important elementary work, whoſe author is ſufficiently known to our readers, than that the ingenuity of the editor, and the liberality of the publiſhers, are equally conſpicuous.

---

*Elements of Chemiſtry; comprehending all the moſt important facts and principles in the works of Fourcroy and Chaptal; with the addition of the more recent chemical diſcoveries which have been made known in Britain and on the Continent; and with a variety of facts and views, which have never before been communicated to the world. Intended for the uſe, not only of thoſe who ſtudy Chemiſtry with thoſe profeſſional purpoſes to which this ſtudy is commonly preferred, but alſo for farmers, manufacturers, dyers, and the other artiſans of the Chemical Arts in general, &c.* By ROBERT HERON. octavo. About 700 pages. Price 12s. 1800. London, Longman and Rees.

THE Author's aim, in the compoſition of the preſent work, appears pretty evident from this circumſtantial title, as well as the preface: He affirms that the *phlogiſtic theory* is ſtill lurking, not

 only only in the very best works of the French chemists, such as those of FOURCROY and CHAPTAL, but "that it triumphs in the chemical writings of most of those *great men*, the STOLIDISSIMI (we apprehend the STAHLIANS) of Germany." He farther censures the *unscientific confusion of arrangement* prevailing in all former systems of Chemistry, except only the "Philosophy of Chemistry," by Fourcroy; while he endeavours to account for this confusion, and to present the world with an arrangement more *scientific* and *logical*, of which the following is an outline:

"The *simple* substances," says Mr. Heron, "at least those which have not been hitherto decomposed, are considered so many distinct CLASSES: *Compounds*, in which two or three of these simple substances exist in union, are regarded as ORDERS: the *compounds* of those compounds constitute GENERA. Subordinate to these are the SPECIES; which are, of course, made up of VARIETIES; and these, of INDIVIDUALS."—"In the whole, he cannot but hope, that the arrangement he has followed will be found to be not only the *most scientific*, but *by much* the best adapted to *open up* the science of Chemistry to the easy intelligence of the Reader's mind."

We shall not, in this place, attempt to investigate and *duly* appreciate Mr. Heron's claims to originality; for, besides his new arrangement, he has also advanced *a new theory of the earth*; and, while he charges Dr. Beddoes and others with a considerable portion of *empiricism* (p. xxii. of the Preface) he informs the reader, that in his book he has endeavoured to DEMONSTRATE, "that *in all the functions of the animal powers*, whether in health or sickness, there intervenes between the agency of *mechanism* and *mechanical causes*—and that of *vitality*—a CHEMICAL AGENCY, the thorough knowledge of which can alone enable us to establish the foundations of true medical science. This doctrine is ENTIRELY NEW in medicine. If true; it is of infinite importance. Of all the applications of chemistry, it must prove the most beautiful and the most interesting. It cannot but confer new dignity on chemical science; since it exhibits it in this new relation to the principal utilities of human life. That the *general truth is fully demonstrated*, the author entertains the strongest confidence: That he may have erred in some of those particular details into which he has attempted to follow it—is *exceedingly* probable. He cannot but hope, that, by the truly candid and philosophical physician, he will be owned to have *opened up* a new path for medical investigation, which, in preference to almost all others, deserves to be instantly and diligently explored," pp. 22 and 23 Preface.

Far from wishing to discourage new adventurers on so arduous and obscure a path of inquiry as that ventured upon by the author of these "Elements;" we would always recommend to writers a certain degree of modesty, and deference to the opinions of others, even on those occasions where our predecessors have obviously erred. No medical reader, we apprehend, will be regulated by the fanciful conjectures of those whose study is to censure, and not to correct or improve; to demolish the old fabric, without erecting a new new one; becaufe they are little concerned about the ftability and folidity of the fuperftructure, provided they fucceed in broaching new theories, or new fancies: — whether fuch fuggeftions be of practical utility, or calculated to explain a fingle fact in Nature, does not appear to be *their* principal object.

To juftify thefe well-meant remarks, we beg leave to refer the reader to the work before us; viz. pp. ii, iii, vii, ix, x, xii, &c. of the Preface. In tracing, however, the merits and originality of Lavoifier, Mr. H. appears to have made a very important difcovery. He informs us, "that he has found, even in Spratt's Hiftory of the Royal Society, an account of a *Theory of Combuftion*, not merely akin to that of *Lavoifier*, but precifely, identically, indubitably the fame; a theory fupported by the indication of a train of experiments, not lefs ample than that of the French chemifts. It is *not poffible* that this Theory, and the experiments indicated for its fupport, fhould have been unknown to the French Academicians. It is aftonifhing that its exiftence, and its coincidence with that of Lavoifier, fhould not have been fooner popularly pointed out."— We are not prepared to examine this extraordinary coincidence; but we truft that our correfpondents, who have taken confiderable pains to afcertain the claims of Mayow and other chemifts, will not fail to inquire into the merits of this curious fubject, and favour us with the refult of their refearches.

In the *fourth Appendix* to this work, of which there are no lefs than *five*, Mr. Heron endeavours to prove, that "*lime is oxygen in a concrete ftate,*" while he informs us, that "*A Dr.* MITCHILL, of New York, amidft fome *very inaccurate* chemical notions, has, *with great juftice,* reprefented the ufe of lime-ftone in paving the ftreets, in building, &c. as tending to prevent the infection of the yellow fever, and of whatever other difeafes originate in a deficiency of gas-oxygen. But it is impoffible that *lime* fhould, by any of its other known qualities, accomplifh fuch an effect,—unlefs by an infenfible converfion of it into *gas-oxygen.*"

---

*A Continuation of Facts and Obfervations, relative to the Variolæ Vaccinæ, or Cow-Pox.* By EDWARD JENNER, M. D. F. R. S. F. L. S. &c. 4to. London, pp. 42. Law, &c.

WE cannot introduce thefe Facts and Obfervations better than in the words of the Author.

"Since my former publications on the Vaccine Inoculation, I have had the fatisfaction of feeing it extend very widely. Not only in this country is the fubject purfued with ardour, but from my correfpondence with many refpectable medical gentlemen on the Continent, (among whom are, *Dr. De Carro*, of Vienna, and *Dr. Ballhorn*, of Hanover,) I find it is as warmly adopted abroad, where it has afforded the greateft fatisfaction. I have the pleafure, too, of feeing that the feeble efforts of a few individuals to depreciate the new practice, are finking faft into contempt, beneath the immenfe mafs of evidence which has rifen up in fupport of it.

"Upwards of fix thoufand perfons have now been inoculated with the the virus of Cow-pox, and the far greater part of them have ſince been inoculated with that of Small-pox, and expoſed to its infection in every rational way that could be deviſed, without effect.

"It was very improbable that the inveſtigation of a diſeaſe ſo analogous to the Small-pox, ſhould go forward without engaging the attention of the Phyſician of the Small-pox Hoſpital in London.

"Accordingly, Dr. Woodville, who fills that department with ſo much reſpectability, took an early opportunity of inſtituting an Inquiry into the nature of the Cow-pox. This Inquiry was begun in the early part of the preſent year; and in May, Dr. Woodville publiſhed the reſult, which differs eſſentially from mine in a point of much importance. It appears, that three-fifths of the patients inoculated were affected with eruptions, for the moſt part ſo perfectly reſembling the Small-pox, as not to be diſtinguiſhed from them. On this ſubject, it is neceſſary that I ſhould make ſome comments.

"When I conſider that out of the great number of caſes of caſual inoculation immediately from cows, which have from time to time preſented themſelves to my obſervation, and the many ſimilar inſtances which have been communicated to me by medical gentlemen in this neighbourhood; when I conſider too, that the matter with which my inoculations were conducted in the years 1797, 98, and 99, was taken from different cows, and that in no inſtance any thing like a variolous puſtule appeared, I cannot feel diſpoſed to imagine that eruptions, ſimilar to thoſe deſcribed by Dr. Woodville, have ever been produced by the ***pure, uncontaminated Cow Pock virus:*** on the contrary, I do ſuppoſe that thoſe which the Doctor ſpeaks of, originated in the action of variolous matter, which crept into the conſtitution with the vaccine. And this I preſume happened from the inoculation of a great number of the patients with variolous matter (ſome on the third, others on the fifth day) after the vaccine had been applied; and it ſhould be obſerved, that the matter thus propagated became the ſource of future inoculations in the hands of many medical gentlemen who appeared to have been previouſly unacquainted with the nature of the Cow-pox.

"Another circumſtance ſtrongly, in my opinion, ſupporting this ſuppoſition, is the following: The Cow-pox has been known among our dairies, time immemorial. If puſtules then, like the variolous, were to follow the communication of it from the cow to the milker, would not ſuch a fact have been known, and recorded at our farms? Yet, neither our farmers, nor the medical people of the neighbourhood, have noticed ſuch an occurrence."

The Author next adduces a number of circumſtances and communications from various practitioners, tending to confirm the preceding opinions. He concludes thus:

"This Inquiry is not now ſo much in its infancy, as to reſtrain me from ſpeaking more poſitively than formerly on the important point of Scrophula, as connected with the Small-pox.

"Every practitioner in medicine, who has extenſively inoculated with the Small-pox, or has attended many of thoſe who have had had the distemper in the natural way, must acknowledge that he has frequently seen scrophulous affections, in some form or another, sometimes rather quickly shewing themselves after the recovery of the patients. Conceiving this fact to be admitted, as I presume it must be by all who have carefully attended to the subject, may I not ask, whether it does not appear probable that the general introduction of the Small-pox into Europe has not been among the most conducive means in exciting that formidable foe to health? Having attentively watched the effects of the Cow-pox in this respect, I am happy in being able to declare, that the disease does not appear to have the least tendency to produce this destructive malady.

"The scepticism that appeared even among the most enlightened of medical men, when my sentiments on the important subject of the Cow-pox were first promulgated, was highly laudable. To have admitted the truth of a doctrine, at once so novel and so unlike any thing that ever had appeared in the annals of medicine, without the test of the most rigid scrutiny, would have bordered upon temerity; but now, when that scrutiny has taken place, not only among ourselves but in the first professional circles in Europe, and when it has been uniformly found in such abundant instances, that the human frame, when once it has felt the influence of the genuine Cow-pox in the way that has been described, is never afterwards at any period of its existence assailable by the Small-pox, may I not with perfect confidence congratulate my country and society at large on their beholding, in the mild form of the Cow-pox, an antidote that is capable of extirpating from the earth a disease which is every hour devouring its victims; a disease that has ever been considered as the severest scourge of the human race."

---

*Observations on the History and Cause of Asthma; and a Review of a "Practical Enquiry on disordered Respiration," in a Letter to Dr. Bree, the author of that work.* By G. Lipscomb, Surgeon at Birmingham, &c. &c. 8vo. pp. 108, price 3 s. London, Johnson.

This work will doubtless be read by many practitioners, and by many who, perhaps, will be no less pleased with the manner than with the matter of it. With respect to our own simple taste, we have no hesitation in confessing that we should have preferred the flavour of the dish, if there had been less pepper and vinegar in the sauce.

---

*The Chemical Pocket-book, or Memoranda Chemica; arranged in a compendium of chemistry, according to the latest discoveries, with Bergman's Table of single elective attractions, as improved by Dr. G. Pearson, &c. By* James Parkinson, 12mo. pp. 230, London, Symonds, &c.

The increasing importance of Chemistry in numerous branches of science, must render a well arranged compendium of its principles, a valuable and acceptable present to the public. The well known skill, accuracy, and industry of the author of this compendium are are sufficient, independent of our own approbation on perusal, to justify our recommendation of it to all students in chemistry. It is, literally, *multum in parvo*. We notice an obvious inaccuracy, not mentioned in the author's list of errata, which occurs at page 34, line 3, "triangular octoedrons of a prismatic figure," &c. *read*, irregular octoedrons &c.

---

*An Essay on the Nature and Connection of Heat, Electricity, and Light. By* A. ANSTRUTHER, Esq. of Madras, Barrister at Law; 8vo. pp. 61. London, Murray and Highley, &c.

THE questions, respecting the source and origin of caloric; the quantity of caloric contained in the direct solar rays; the sudden change of temperature which takes place during hail storms; and the destination and reproduction of that heat which is evolved during the condensation of vapour into rain or hail, are involved in much obscurity.—It is the object of this ingenious Essay to answer these questions; and though we are not convinced that the author has entirely succeeded in his attempts, we think him intitled to the apology, "Magnis tamen excidet ausis."

---

*A Short Introduction to the Knowledge of Gaseous Bodies. By Dr.* A. N. SCHERER, &c. translated from the German, 8vo. pp. 110. London, Treppas, &c.

THIS may be considered as a compendious Text-book of the learned Professor's Course of Lectures, on the first Principles of Chemistry, read to a popular audience; similar to the courses delivered at the Royal Institution in Albermarle Street. We are well assured that the lovers of Chemisty will not fail to peruse this short pamphlet, independent of our recommendation.

---

*Elements of Botany, illustrated by Engravings.* By JOHN HULL, M. D. &c. in Two Vol. 8vo. pp. 700. Manchester, Dean. London, Bickerstaff, &c.

THE first volume contains an elaborate introduction to the Linnæn system, with a very extensive explanation of Botanical terms, illustrated by plates, natural orders, indices, and an alphabetical dictionary, which includes the systems of other Botanists.

The second vol. contains an enumeration of all the genera of British plants, and an explanation of several designs for a natural order, with the necessary indices. We consider this second volume as the best pocket companion for the Tyro, in Botanical excursions, that has fallen under our notice.

---

*Ideen zu einer Philosophie der Natur.* Ideas, or Outlines towards a Philosophy of Nature. By F. W. J. SCHELLING, 8vo. Two Volumes. *Leipzig*, Breitkopf and Härtel.

IN this profound work we discover a spirit of inquiry, which pursues its aim with peculiar energy, and in its progress affords many many luminous views of the furrounding, though remote objects; but which may, with more juftice, be called original, than fundamental.

In the Introduction, the author propofes the different problems which ought to be folved by a Philofophy of Nature. The exiftence of Nature, that is, of the whole experimental world, fhould be derived from principles, and thus would be eftablifhed a fcientific fyftem of phyfics. But we are accuftomed to conceive a determined feries of phenomena, as a neceffary confequence of caufes and effects; and all our experimental Sciences, Natural Philofophy, and even Hiftory itfelf, are founded upon thefe conceptions. A feries, or fucceffion of ideas, however, is a fomething, the poffibility of which exifts only in the reprefenting capacity of the mind; our choice, therefore, is limited to the following alternative: *Either*, we maintain that things exift externally to us, independent of our reprefentations; and in fuch cafe we explain the neceffity with which we reprefent to ourfelves a determined feries of things as a mere illufion, by denying that the fucceffion takes place in the things themfelves; *or*, we admit, that the phenomena themfelves, together with the idea of fucceffion, originate in our reprefentations alone; and that fo far only the order in which they follow each other, is truly an object of fenfe. The former affertion leads to the moft abfurd fyftem that ever exifted, fo that the fecond method alone can be admitted. Here, therefore, in the abfolute identity of the mind within us, and in Nature without us, the great problem muft be folved, how fuch a thing as Nature can poffibly exift externally to us. Nor is there any other practicable method of folving the queftion. For we do not require to know, how a Nature has originated which is external to the human mind; but our object is to difcover only how we have acquired the idea of fuch a Nature; and this not merely refpecting the manner in which we have fpontaneoufly or arbitrarily conceived it, but the reafons why, and how it conftitutes the original and neceffary bafis of every thing that has yet engaged our attention, when reflecting on the exiftence of that Nature.

We have, in the preceding paragraph, given our readers a fpecimen of the reafoning adopted in this metaphyfical work: we prefume, however, it will be more fatisfactory to them, when we point out its general contents, together with fome new opinions and illuftrations peculiar to the author.

In the firft volume, M. Schelling treats of the combuftion of bodies, of light and heat, of air, electricity, and the magnet.

In the fecond volume, the author makes fuch reflections as relate more to the Syftem of Nature in general than to particular phenomena. He endeavours to derive the principle of attraction and repulfion from a different and more profound fource than Newton's innate powers of matter; for, in the opinion of the former, it is the condition of the poffible exiftence of matter itfelf; or rather, that matter confifts of nothing elfe than thofe powers, when conceived in oppofition to each other.

The Philofophy of Chemiftry, difcuffed in the Seventh Chapter, as an experimental fcience, the object of which is to inquire into the qualitative difference of matter, and the refpective attraction and repulfion arifing therefrom, lead the author to conclude, that all qualities of matter folely and exclufively depend upon the intenfity of their original powers.

---

*Alexander Monro's Abbildungen, &c.* Alexander Monro's Defcription of the Burfæ Mucofæ of the Human Body, tranflated into German and Latin, with improvements and additions: By Dr. J. C. Rosenmuller, Diffector to the Anatomical Theatre at Leipzig; folio, with 15 plates; price 10 dollars. Leipzig, Breitkopf and Hartel. 1800.

This work is not a mere Tranflation of Dr. Monro's book, which was publifhed at Edinburgh in the year 1788, but an edition improved throughout, and containing fuch a number of new difcoveries and illuftrations, as to give it a title amongft the original works of fcience. Not only the anatomy of all the Burfæ Mucofæ is here explained, but alfo the Phyfiology and Pathology. The doctrine of the Burfæ Mucofæ, upon which we were hitherto only in poffeffion of fome fragments (among which the original work of Monro is to be reckoned), we find here delivered in a fyftematical order, which connectsit with the other doctrines of Anatomy, that have already been treated according to the fame method.

Befides the plates of Monro, which have all been corrected according to comparifons with the real fubject, fo as to render them much more intelligible than thofe of the original, Dr. Rofenmuller has added engravings of the burfæ mucofæ of the head and trunk, which were not known to Monro, when he firft publifhed his work. Thus the whole fyftem of thefe organs is completed, and prefented to the reader in one connected view.

The text of Monro has no lefs undergone a thorough revifion and new elaboration. All the Burfæ Mucofæ, which he has only curforily mentioned in the explanations annexed to his plates, are here accurately examined and defcribed. The defcriptions of feveral Burfæ difcovered by the author, which were hitherto unknown to anatomifts, are added, and the accounts given by other authors of their own inveftigations into this fubject, are inferted in their proper places. The phyfiological difquifitions, into the nature of the fecretion performed by thefe organs, and the well arranged enumeration of the difeafes to which they are fubject, their caufes, diagnofis, and indications of cure, with which Dr. R. has enriched his work, render it the moft complete and inftructive treatife extant upon the fubject.

The firft Section contains a complete catalogue of all the treatifes that have hitherto appeared upon the Burfæ Mucofæ, arranged according to the order of their publication. The plates are correctly engraved after Dr. Rofenmuller's own drawings.

A Curfory

*A Curfory View of the Treatment of Ulcers, more efpecially thofe of the Scrophulous, Phagedenic, and Cancerous Defcription, with an Appendix on Baynton's new Mode of treating Old Ulcers of the Leg.* By RICHARD NAYLER, Surgeon to the Gloucefter Infirmary, pp. 180. London, Kearfley.

THIS pamphlet is written with perfpicuity, and contains obfervations on reft, and horizontal pofition,—on internal remedies,—on conftitutional complaints accompanying ulcers of the leg,—on the fymptoms of ulcers, and on topical remedies. When fpeaking on fomentation, the author juftly remarks, that "the degree of heat is a circumftance not often attended to, but the practice too commonly followed, is that of applying it as *hot* as the patient can bear." This the author obferves, muft be frequently very injurious. "It is probable, that the irritable ulcer would be particularly liable to fuffer by it, for the degree of heat, acting as a violent ftimulant, muft, of courfe, be difadvantageous where every thing ftimulating is contra-indicated."

As a dreffing for large ulcers, the author recommends the ufe of tow in preference to lint, as being more *pervious* to the matter difcharged from it; but we truft that his cenfure on the mode of drefsing ulcers in the London Hofpitals, is much too general to be correct. "As the application of dreffings, and of the bandage, as far as the *manner* of doing them is concerned, ufually falls under the management of gentlemen, fcarcely yet initiated in chirurgical bufinefs, it is too common to fee them haftily, and of courfe, inadequately, performed; and it is particularly unfortunate, that the hurrying way in which ulcers are dreffed in the London Hofpitals, affords the ftudent fo few opportunities of convincing himfelf, how effential to the cure of an ulcer is a *deliberate, neat, and fyftematic* way *of applying the neceffary remedies.*"

The author proceeds with obfervations on ulcers, in the production of which the conftitution participates,—the fcorbutic, the venereal, the fcrophulous, the phagedenic, and the cancerous ulcers. On the laft fpecies, when it attacks the uterus, the author hazards a conjecture, which, whether true or not, deferves our ferious confideration. "Is there not a probability that the practice of ignorant midwives, of dilating the mouth of the womb during labour, by which it may be fairly prefumed, laceration fometimes happens, is among the caufes which occafion cancer?"

In the Appendix, the author differs in opinion with Mr. Baynton, as to the modus operandi of his invention, and thinks "the application of the auxiliary remedy, cold water, of almoft equal importance with the principal, and he hefitates to admit its claim to *uniformity of fuccefs*, even in the *faireft* cafes that can occur for the experiment," although he allows it to have had an *ample fhare* of fuccefs in various cafes under his care at the Infirmary.

---

*Plain and ufeful Inftructions for the Relief and Cure of Ruptures, &c.* By J. EDDY, M. S. D. pp. 40. Symonds, London.

Mr. Eddy is a trufs maker, and refides at No. 43, Dean-ftreet, Soho, where he profeffes to cure Ruptures by his *Patent Perizoma!*

*Botanique pour les Femmes, &c.*—Botany for the Ladies and Amateurs of Plants; by R. J. G. Ch. Batsch, M. D. Professor at Jena; with 101 coloured figures; translated from the German into French, and augmented with Notes and other Additions, by J. E. B***, Associate of the National Institute in France. Paris and Strasburg, 7th year. 8vo. 198 pages.

The translator has not only given a faithful translation of this work, but has often found himself under the necessity of developing the author's ideas, of illustrating them by examples, and sometimes of embellishing them in a more gay or sentimental stile, in order to prove that he never lost sight of the sex for whose amusement the work is chiefly adapted. M. Batsch wrote for Germans, who do not always require an embellishment of words, but wish a serious work to be treated with gravity. Cit. B. conceived, that in writing for French females, he ought to adorn the work with the charms of imagination, and even borrow, without affectation, the language of the passions.

---

*Lettres du Docteur* William Kentish, *neveu de Smellie, au Cit.* Baudelocque, *&c.*—Letters from Dr. William Kentish, nephew of Dr. Smellie, to Cit. Baudelocque, relative to some Passages in his Treatise on Midwifery. Paris, in the 8th year.

Those who profess a liberal impartiality in the study of midwifery, will, with great satisfaction, read the discussions entered into by the author, with a view to excite attention to some errors which, in his opinion, have escaped Cit. Baudelocque.

*Non licet inter vos tantas componere lites.*

We shall only observe, that if the criticisms contained in these letters are not the most accurate, they are couched in a free and delicate language.

Besides, many theoretical and practical points are here much better illustrated than in any other work of the kind. It is from this motive we recommend their perusal to all accoucheurs.

---

N. D. Riegels *Philosophiæ animalium, fasciculus primus, de Erinaceo, &c.*—Riegels's Philosophy of Animals. Fascicle 1, on the Hedge-hog, detailing its organs of digestion, chylification, secretions from its parts of generation, its osteology, muscles, instinctive powers, &c. and with various Physiological Problems. Copenhagen, 1799. 12mo. 32 pages.

In the Introduction, the author gives an historical account of the knowledge of the ancients in zoology, and of the works written by them on this subject; he developes some principles established by Aristotle, and defends this author against some attacks made on him by Bacon. He then proceeds to a description of the *Hedge-hog*, and begins with enumerating the organs of digestion and chylification, those of generation and of life; he then treats of the osteology of this mammiferous animal, of its manners, instinct and senses.

After having stated what is already known of the hedge-hog, he concludes with some problems relative to that animal, and a few observations on the use we make of it in medicine and common life. M. Riegels intends successively to treat in the same manner on rats, the phocas, the mole, the frog and lizard, the swine, the sheep, the hare, the fowl, the duck and the goose, the crow, the dog, &c. He likewise intends to point out the different uses of each of these animals. This first fasciculus is also separately sold under the title of *Scrutatio anatomico-philosophica de Erinaceo, auctore* N. D. Riegels.

---

Gerardi Vrolik, *Oratio de viribus vitalibus, &c.*—An Oration, by Gerard Vrolik, on the Vital and constant Powers observable in every organic Substance; delivered in November, 1798, upon the occasion of investing the Professorship of Anatomy, Physiology, and the Obstetric Art, at the Athæneum of Amsterdam, 1799. 4to. 44 pages.

In this speech, delivered with the view of obtaining the chair of anatomy, physiology, and midwifery, in the College at Amsterdam, M. Vrolik treats of a truly interesting subject, perfectly corresponding with the sciences he teaches. It would appear almost impossible for the author, entirely to exhaust this vast matter within the narrow limits of a speech; he has not been able to give more than a short outline of his plan, and briefly to treat on its principal heads; in which he has proved to his auditors, that time has not allowed him to enter more largely into his subject.

---

*An Inquiry into the Symptoms and Causes of the Syncope Anginosa, commonly called Angina Pectoris; illustrated by Dissections.* By C. H. Parry, M. D. &c. 8vo. pp. 169. Price 4s. Bath, Crutwell; London, Cadell and Davis.

We are much pleased to observe, that a disease so recently distinguished by practitioners, so obscure in its symptoms, and so fatal in its progress, should have excited the attention of a physician so capable of illustrating its pathology.

Dr. P. in the Introduction, informs his readers, that "The substance of the following Essay was originally read to a Medical Society in Gloucestershire. This little Society consisted of the following persons: Dr. Hickes, of Bristol; Dr. Jenner, of Berkeley, in Gloucestershire, well known to the public by his ingenious paper on the Cuckoo, and by his original communications on the important subject of the Cow-pox; Dr. Ludlow, of Corsham; Mr. Paytherus, of the Adelphi, London; and the Auhor of these pages."

Dr. P. then details a number of cases mentioned by other authors, and several which had fallen under his own notice and that of of the gentlemen juſt mentioned, with the appearances on diſſection. From theſe data he gives the following enumeration of

SYMPTOMS.

" The firſt ſymptom is an uneaſy ſenſation, which has been variouſly denominated a ſtricture, an anxiety, or a pain, extending generally from about the middle of the ſternum acroſs the left breaſt, and, in certain ſtages of the diſorder, uſually ſtretching into the left arm, a little above the elbow. In ſome few examples, the pain, ſtricture, or anxiety, is in a certain degree felt alſo acroſs the right breaſt; and occaſionally, though I believe rarely, has extended itſelf to one or both wriſts.

" The pain which I have deſcribed, occurs in paroxyſms, and, in the early periods of the diſeaſe, is ſeldom produced without ſome apparent cauſe, ſuch as walking, particularly up hill or up ſtairs, againſt the wind, or in a quick pace. On theſe occaſions, the patient feels as if perſiſting in the exertion would produce a total ſuſpenſion of the powers of life. He therefore ſtands ſtill, or turns from the wind; on which the uneaſy ſenſation ſoon vaniſhes. We are told of one patient, who appears to have been, in other reſpects, a man of unuſual firmneſs of mind, that he had the reſolution to continue walking, and that he found the pain go off after it had affected him from five to ten minutes. This ſenſation in the breaſt often admits of temporary relief from the evacuation of wind by the mouth, and is altogether ſo free and diſtinct from any difficulty of breathing, that patients during the paroxyſm make a deep inſpiration with the utmoſt eaſe, and, in ſome inſtances, appear to to be fond of ſighing deeply, and of retaining their breath. In ſome caſes, it is either conjoined with an unequal pulſe, or affects perſons who are ſubject to that ſymptom. In other caſes, the pulſe has been habitually ſo little changed, as to lead to the opinion that the heart in no reſpect primarily ſuffers. But whatever may be the ſtate of the pulſe as to regularity, I believe we ſhall always find it become more or leſs feeble according to the violence of the paroxyſm.

" In the ſlighter caſes, and in this firſt ſtage of the diſorder, the fit ſeldom comes on but from the exertions which I have mentioned; and as it is probable that experience of their miſchievous effects will cauſe theſe exertions to be as much as poſſible ſhunned, patients will continue many days, and ſometimes weeks, without any attack of the diſeaſe. It has been obſerved, that paroxyſms are moſt apt to occur from walking after a meal. In general, they are not excited by exerciſe on horſeback, or in a carriage, or by ſome ſhort and partial though ſtrong exertions of the body itſelf, as in talking, laughing, coughing, or vomiting. They have been by ſome thought to occur moſt frequently in the extremes of hot and cold weather; but in many inſtances, there has been no perceptible difference in this reſpect.

[ To be concluded in our next Number. ]

